# A look into the future of blockchain technology

**DOI:** 10.1371/journal.pone.0258995

**Published:** 2021-11-17

**Authors:** Daniel Levis, Francesco Fontana, Elisa Ughetto

**Affiliations:** 1 Groupe ALTEN, France; 2 Politecnico di Torino, Corso Duca degli Abruzzi 24, Turin, Italy; 3 Politecnico di Torino & Bureau of Entrepreneurial Finance, Corso Duca degli Abruzzi 24, Turin, Italy; University of Salento, ITALY

## Abstract

In this paper, we use a Delphi approach to investigate whether, and to what extent, blockchain-based applications might affect firms’ organizations, innovations, and strategies by 2030, and, consequently, which societal areas may be mainly affected. We provide a deep understanding of how the adoption of this technology could lead to changes in Europe over multiple dimensions, ranging from business to culture and society, policy and regulation, economy, and technology. From the projections that reached a significant consensus and were given a high probability of occurrence by the experts, we derive four scenarios built around two main dimensions: the digitization of assets and the change in business models.

## 1 Introduction

Over the last few years, the hype and interest around blockchain technology have consistently increased. Practitioners from many industries and sectors have joined an open, yet mainly unstructured, discussion on the potential disruptive capabilities of this newly born technology [1–3]. In principle, the size of the phenomenon could be huge, with latest estimates predicting blockchain to store, by 2025, the 10 per cent of the world’s GDP (about $88tn in 2019) [4]. However, the complexity of the technology itself and the difficulties in assessing its impact across the different application fields have prevented the social, industrial and scientific communities to agree upon a shared vision of future blockchain-based scenarios. Very fundamental questions are still to be answered. Which blockchain-enabled applications will see the light in the next few years? Which industrial sectors will be mainly affected? How will companies react to potential industry-disruptors? How will the current societal paradigm shift? Which role will policy makers play in enhancing this new paradigm?

Despite the great and undoubted technological innovation linked to this technology, uncertainties and speculation on the potential scenarios still animate the industrial and scientific dialogue [5]. In particular, it is not yet clear which applications will see the light, and, eventually, what effects these changes will have at a societal level.

In this paper, we use a Delphi approach to investigate whether, and to what extent, blockchain-based applications will affect firms’ organizations, innovations and strategies by 2030, and, consequently, which societal areas will be mainly affected. With this methodology, we aim at reaching experts’ consensus to gain new insights and assess the likelihood about the future of the technology. This is a relevant issue, as blockchain technology applications cover a wide spectrum of areas. Blockchain can be applied vertically within an industry (e.g. disrupting its supply chain) or horizontally across different industries or within single companies (e.g. modifying the internal structures and the modus operandi of the different company functions). Given the number of potential applications and the complexity of the technology, stakeholders are divided into skeptics, who believe the technology is still too immature to become a paradigm in the near future, and enthusiasts, who instead believe that this radical innovation will disrupt many industries and completely change business models and people’s behaviors, like internet did during the 90s.

The literature on blockchain is also widely fragmented. Different works have investigated possible blockchain applications within specific domains, such as finance [6–8], logistics [9], healthcare [10, 11] and education [12]. However, a holistic approach on possible blockchain-enabled future scenarios is still missing. To our knowledge, the only contribution in this direction is the one by White [13], who explores blockchain as a source of disruptive innovation exclusively with regard to the business field. We depart from his work to adopt a much wider perspective in this study. In fact, our aim is to obtain a deep understanding on how the adoption of this technology in Europe will lead to changes over multiple dimensions, ranging from business to culture and society, policy and regulation, economy and technology. Thus, our research aims at exploring if a convergence between the two divergent perspectives on blockchain can be found, bringing together experts currently working on blockchain projects to explore the possible changes that the technology will bring to the society by 2030.

Our study outlines an overall agreement among experts that the blockchain technology will have a deep impact on multiple dimensions. In the near future people will likely start using and exploit the blockchain technology potential, without really knowing how the technology behind works, in the same way as they send emails today, ignoring how the digital architecture that allows to exchange bytes of information works. Policy makers and governments will play a crucial role in this respect, by enabling productivity boosts and competitive gains from the companies operating under their jurisdictions. As such, a tight and cooperative relationship between industrial actors and regulatory bodies will be extremely important and auspicial. To this aim, it will be of key importance for all players to understand the real competitive advantage that blockchain can bring to their own industry and market.

This work aims at contributing to the raising blockchain literature by offering a holistic view on possible blockchain-enabled future scenarios in Europe, and to investigate which of the proposed scenarios is more likely to occur. As widely agreed by the academic literature, technological developments dictate the speed and pace at which societies change [14]. Under this assumption, technological forecasting appears to be a method of fundamental importance to understand “ex-ante” the potential development of technological changes, and their impact on different societal aspects [15]. Foreseeing future technological trends could help society in understanding possible future scenarios, thus contributing to a better knowledge of the new paradigms our society is heading towards. The work is structured as follows. Section 2 provides an overview on the main research streams upon which this work is based. Section 3 presents the methodology. Results are described in Section 4 and Section 5 concludes the work.

## 2 Background literature

### 2.1 The blockchain technology

As defined by Crosby et al. [3] a blockchain can be conceptualized as a shared and decentralized ledger of transactions. This chain grows as new blocks (i.e. read transactions or digital events) are appended to it continuously [16, 17]. Each transaction in the ledger must be confirmed by the majority of the participants in the system [3, 18–21]. This means for the community to verify the truthfulness of the new piece of information and to keep the blockchain copies synchronized between all the nodes (i.e. between all the participants to the network) in such a way that everybody agrees which is the chain of blocks to follow [19]. Thus, when a client executes a transaction (e.g. when it sends some value to another client), it broadcasts the transaction encrypted with a specific technique to the entire network, so that all users in the system receive a notification of the transaction in a few seconds. At that moment, the transaction is “unconfirmed”, since it has not yet been validated by the community. Once the users verify the transaction with a process called mining, a new block is added to the chain. Usually, the miner (i.e. the user participating to the verification process) receives a reward under the form of virtual coins, called cryptocurrencies. Examples of cryptocurrencies are Bitcoins, Ether, Stellar Lumens and many others. Virtual coins can then be used on the blockchain platform to transfer value between users [17–19].

Thanks to a combination of mathematics and cryptography, the transactions between users (i.e. exchange of data and value), once verified by the network and added to the chain, are “almost” unmodifiable and can be considered true with a reasonable level of confidence [17, 19, 22]. These attributes of the technology make it extremely efficient in transferring value between users, solving the problem of trust and thus potentially eliminating the need of a central authority (e.g. a bank) that authorizes and certifies the transactions [7, 23, 24].

The technology can be easily applied to form legally binding agreements among individuals. The digitalized asset, which is the underlying asset of the contract, is called token. A token can be a digitalized share of a company, as well as a real estate property or a car. Through the setting of smart contracts (i.e. digitalized contracts between two parties), the blockchain technology allows users to freely trade digital tokens, and consequently to trade their underling physical assets without the need of a central authority to certify the transaction (OECD, 2020).

### 2.2 Blockchain technology applications

The academic literature has investigated a wide range of possible blockchain applications within specific domains, such as finance [6–8], logistics [9], healthcare [10, 11] and education [12].

As mentioned, one of the undoubted advantages of the blockchain technology is the possibility to overcome the problem of trust while transferring value [25]. Not surprisingly, the technology seems to find more applications in markets where intermediation is currently high, like the financial sector, and in particular the FinTech sector, that has recently experienced a consistent make-over thanks to the diffusion of digital technologies [7, 26, 27]. The implementation of the blockchain technology in the financial markets could provide investors and entrepreneurs with new tools to successfully exchange value and capitals without relying on central authorities, ideally solving the problem of trust. This is among the reasons why many observers believe that the blockchain would become a potential mainstream financial technology in the future [28]. Blockchain represents an innovation able to completely remodel our current financial system, breaking the old paradigm requiring trusted centralized parties [6–8]. With new blockchain-based automated forms of peer-to-peer lending, individuals having limited or no access to formal financial services could gain access to basic financial services previously reserved to individuals with certified financial records [29]. Indeed, blockchain technology can provide value across multiple dimensions, by decreasing information asymmetries and reducing related transactional costs [30]. Initial coin offerings (ICOs) represent one of the most successful blockchain-based applications for financing which has been currently developed. Virtual currencies like Bitcoins can disruptively change the way in which players active in the business of financing new ventures operate [7, 30–33]. Through an ICO, a company in need of new capital offers digital stocks (named token) to the public. These digital tokens will then be used by investors to pay the future products developed by the financed company [30, 34, 35]. ICOs represents a disruptive tool: entrepreneurs can now finance their ventures without intermediaries and consequently lower the cost of the capital raised [31, 36]. However, some threats coming from the technology adoption can also be identified, as blockchain can also lead to higher risks related to the lower level of control intrinsically connected to the technology, especially in the case of asymmetric information between the parties involved.

Disintermediation plays a key role in the healthcare sector as well, where blockchain has recently found numerous applications. Indeed, many players currently need to exchange a huge amount of information to effectively manage the whole sector: from hospitals, to physicians, to patients. The ability to trustfully exchange data and information becomes of undoubted value in this context [10, 11]. It should not be difficult to envision blockchain applications in other fields as well. In every sector in which information, value, or goods are supposed to flow between parties, blockchain can enable a trustful connection between the players, with the need of a central body entrusting the transaction. Within supply chain, it can increase security and traceability of goods [9, 37]. Within education, it can help in certifying students’ acquired skills, reducing, for example, degree fraud [12]. To conclude, a recent work from Lumineau et al. [38] highlights possible implications of the technology in the way collaborations are ruled and executed, shading light on new organizational paradigms. Indeed, the authors show how the intrinsically diverse nature of the technology could strongly affect organizational outcomes, heavily influencing and modifying (possibly improving) the way in which different entities cooperate and collaborate.

## 3 Research methodology

### 3.1 Forecasting technique: the Delphi method

In the past decade, an increasing number of forecasting techniques has been employed in the academic literature to predict the potential developments induced by technological changes. In particular, the Delphi method, whose term derives from the Greek oracle Delphos, is a systematic and interactive method of prediction, which is based on a panel of experts and is carried out through a series of iterations, called rounds. Many academic works have adopted this method since its development [14, 39–44]. As the core of the Delphi approach, experts are required to evaluate projections (representations of possible futures) and assess their societal impact and the likelihood that they will occur within a specific time horizon.

**Fig 1:**
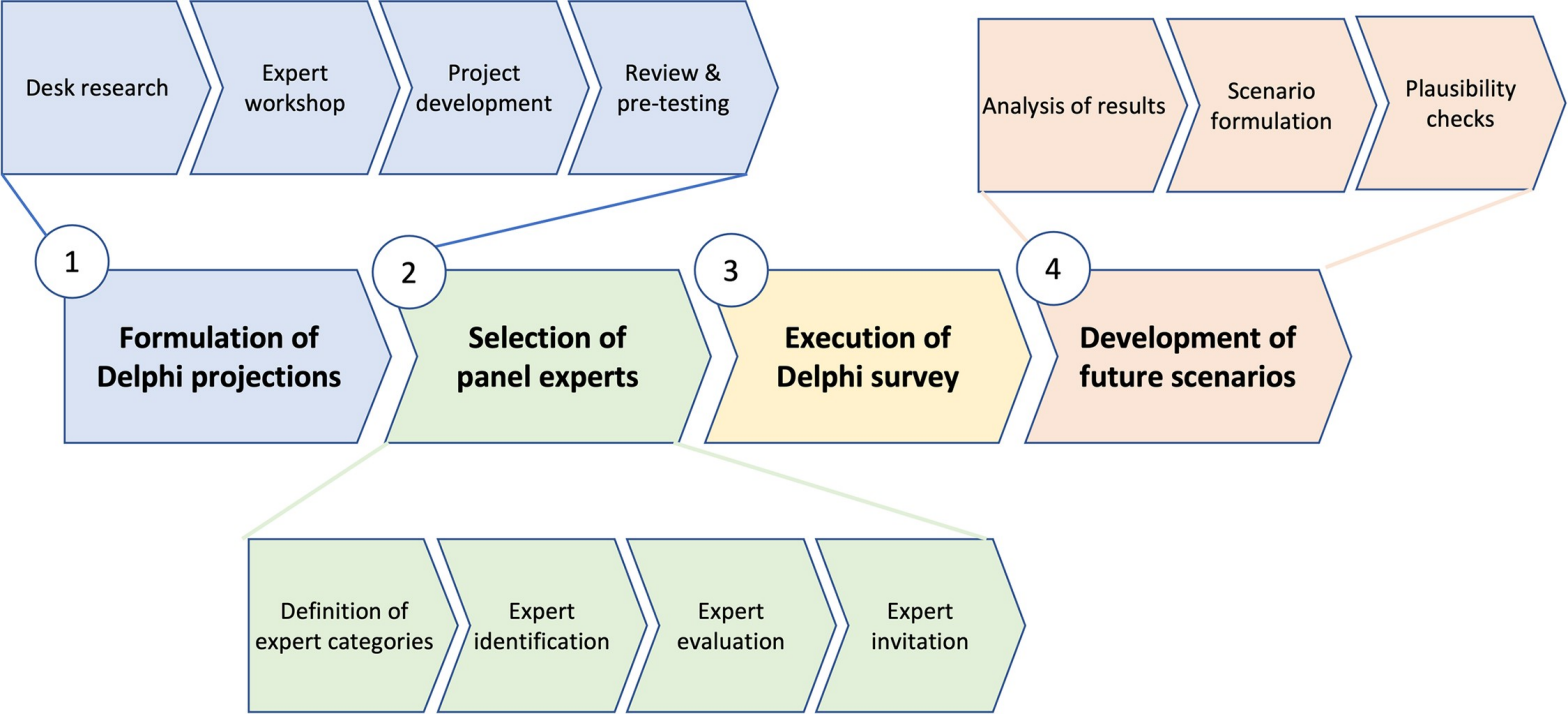
Four Steps Delphi procedure by Heiko and Darkow (2010).

While the majority of forecasting methods does not account for the technological implications on the social, economic and political contexts, the Delphi technique allows subjective consideration of changes in interrelated contexts [45]. Many different variants of the Delphi methodology have been developed according to the needs and goals of each research. For the purpose of this research, we decided to follow the four-steps procedure suggested by Heiko and Darkow [46] (Fig 1).

**Fig 2:**
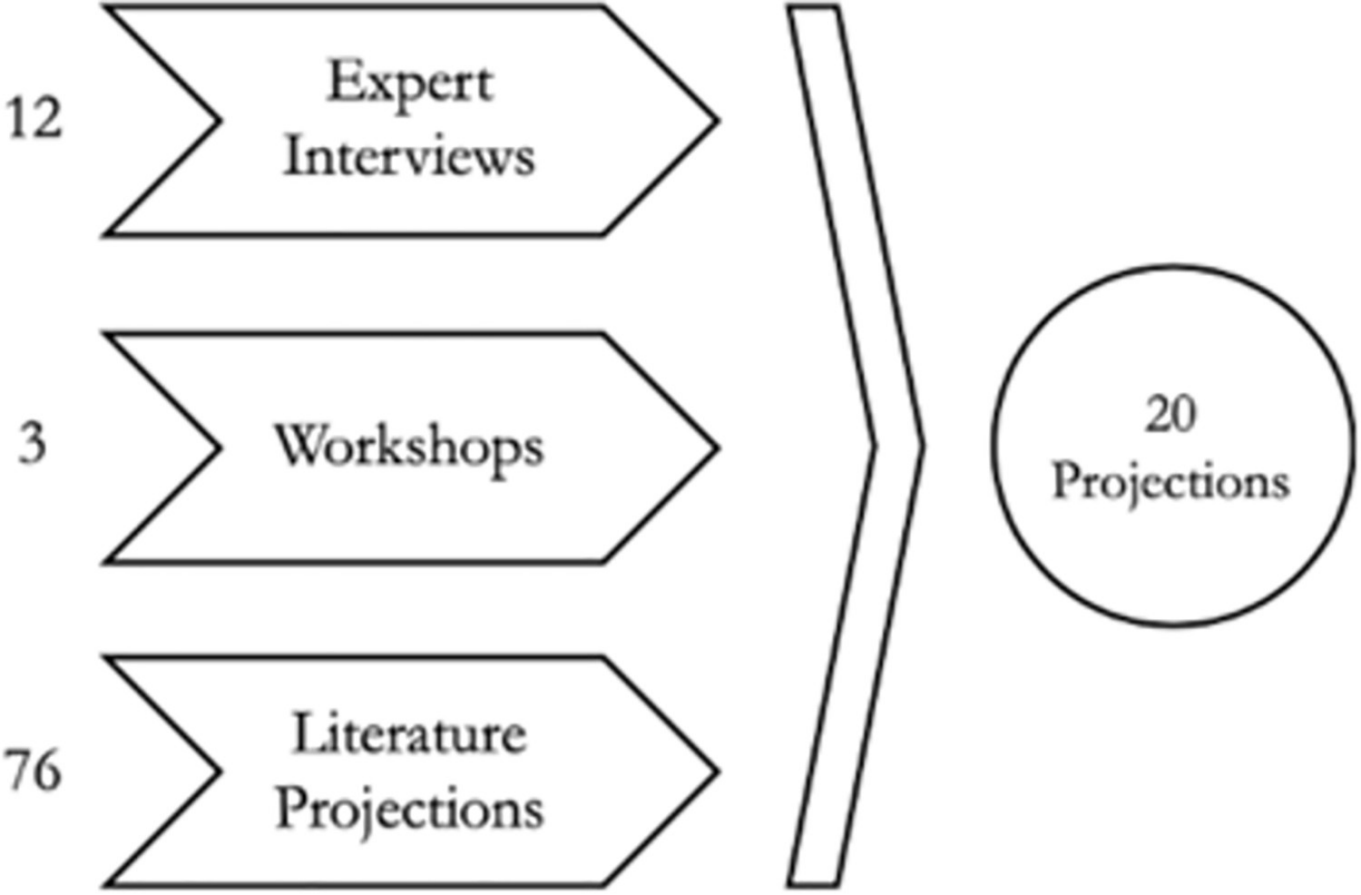
Process followed to generate the final Delphi projections.

The first step of the method requires to develop and envisage projections and possible scenarios that might arise through the adoption of the technology. These projections must be short, unequivocal, and concise [14]. This phase requires researchers to deeply understand the technology by analyzing the existing literature, attending courses and workshops and conducting a number of face-to-face interviews with experts (Fig 2). Once the insights are gathered, the results are synthetized in future projections that will help develop the survey. The second step consists in presenting the study to the panel of selected experts who will take part in the first round of the survey. The main challenge during this phase is to select an appropriate panel of experts and maintain their commitment and response rate. The third step consists in a statistical and quantitative analysis of the answers received and in the selection of the second-round scenarios that experts will need to evaluate again. Through the analysis of the second round of answers, updated scenarios are developed adding to the projections the qualitative and quantitative insights provided by the research. The ultimate goal of this iterative process is to reach consensus among the experts on the scenarios that are most likely to happen in the future.

### 3.2 Formulation of the Delphi projections

The formulation of the projections represents a key aspect of the methodology and requires a particular attention and effort. In this phase, the projections that are later tested by the panel of experts are generated. Vagueness and inaccuracy might generate confusion in experts, leading to less meaningful results. To avoid this situation, we developed the projections by means of triangulation: literature review, interviews with experts and participation to workshops and conferences. The analysis of the literature on blockchain technology (and its benefits) allowed us to understand which industries and businesses will be mainly impacted by the technology.

We chose 2030 as a time horizon for the generation of the scenarios. This is a recommended time span for a Delphi study, since a superior period would have become unmanageable to provide relevant advice for strategic development. As reported in Table 1, projections span among different areas. To the scope of the work, i.e. to grasp a holistic view of the most likely scenarios, it was necessary to investigate a number of multiple dimensions. Projections are related to socio-cultural, policy and regulations, economic, technological and business aspects. As it can be noticed, projections are all structured in the same way, to facilitate their understanding by experts.

**Table 1:** Final 20 Delphi projections.

*No*.	*Projections*
*** ***	**Socio Cultural**
**1**	By 2030 in the European Union, most people and companies will have a degree of knowledge about what a blockchain-based system is and how it works
**2**	By 2030, the European Union will be a leader hub and an example for companies working with blockchain-based technologies, applications and usage
** **	**Policy and Regulations**
**3**	By 2030 in the European Union, blockchain-based technologies will be widely used in order to increase security regarding transactions and data management, as well as to reduce costs and duration of processes
**4**	By 2030 in the European Union, challenges in terms of standards and governance, personal data protection and digital identity management will be solved in order to ensure fair and secure access to data stored on blockchain-based technology
**5**	By 2030, regulations and directives made by the European Union Commission will foster the implementation, innovation and development of blockchain-based technologies and solutions
** **	**Economic**
**6**	By 2030 in the European Union, blockchain-based systems will not eliminate the need for financial intermediaries; they will create a substitution of traditional intermediaries that will require fewer regulations
**7**	By 2030 in the European Union, blockchain entrepreneurship will be focused on designing financial credit services aiming at improving lending practices around efficiency, efficacy and security
**8**	By 2030 in the European Union, most financial services providers will need to radically change their business model in order to adapt to the innovation brought by blockchain-based systems both in terms of infrastructure and services provided
**9**	By 2030 in the European Union, companies that will digitize/ tokenize their assets via blockchain-based systems will have a competitive advantage and will benefit from a higher growth that those who will not implement it
** **	**Technological**
**10**	By 2030 in Europe, blockchain-based technologies will be commonly used and implemented to trace transactions to the financial statement and for other auditing purposes
**11**	By 2030 in the European Union, blockchain-based technologies will enhance the reliability of credit systems, enabling them to adopt tamper proof algorithmic executions
**12**	By 2030 in the European Union, blockchain-based technologies will enable startups and SMEs to have access to loans without the need to provide collaterals
**13**	By 2030 in the European Union, blockchain-based technologies will allow to issue and transfer equity shares on the private exchange marketplaces, by replacing the current paper certificates’ system
**14**	By 2030 in the European Union, ICOs will be commonly used as a way to finance a project, but they will be subject to strict regulations that will ask for many details, such as the code source and the type of tokens issued
**15**	By 2030 in the European Union, most transactions (e.g. payments, property exchanges) will be carried out through blockchain-based systems to ensure reliability and transparency
** **	**Business**
**16**	By 2030 in the European Union, major blockchain applications will be private, among consortiums and company agreements
**17**	By 2030 in the European Union, public blockchains will remain for cryptocurrencies as a form of capital investment
**18**	By 2030 in the European Union, thanks to blockchain-based systems, companies will have access to the digitization of their shares and will be allowed to issue tokenized bonds
**19**	By 2030 in the European Union, blockchain tokens will allow more and more open-source projects to raise funds and support continued development by repaying the developers contributing to the project
**20**	By 2030 in the European Union, smart contracts will be highly adopted for trust-less transactions in financial and economic markets, also extended to stocks, bonds, futures, loans, mortgages, property rights, intellectual property and other contracts

#### 3.2.1 Interviews with experts

Twelve blockchain experts were interviewed among academics, startups’ founders and professionals working in consultancy firms, banks and legal institutions. The selection of the experts was made in order to get different points of view and a high level of expertise, as provided by the Delphi method guidelines. We conducted interviews that took between thirty and forty-five minutes on average, according to the interviewee’s availability. Each single interview was tailored for each participant by providing guidelines and reflection tips to encourage discussion. However, a certain degree of freedom was given to the expert to allow his/her spontaneous contribution and to gain some original insights that helped in the final design of the future scenarios. Some common aspects were discussed in all interviews generating redundancy and repetition of already emerged scenarios (e.g. ICOs, business model evolution, security and utility tokens, and legal issues). This is one of the reasons why twelve interviews were considered to be sufficient for the purposes of our research.

#### 3.2.2 Conferences

One of the authors attended three main events in order to strengthen the knowledge about blockchain and have a broader view of its implications in different fields and industries: one in Milan and two in Paris. Of particular notice, the Community Blockchain Week, a blockchain tech-focused initiative organized voluntarily by actors engaged into the technology and with the will and vision to spread the knowledge among citizens. Thanks to various workshops and speeches during the week, it was possible to dive deeper into many aspects of the technology, as well as to meet some knowledgeable experts of various fields, some of which agreed in participating to the research. The event was extremely useful not only to understand how the technology is evolving, but also to see how the community engages itself to spread the knowledge in order to generate more and more interest around it.

#### 3.2.3 Desk research

We performed desk research to formulate the initial set of projections. Through the survey of the literature, we gained a comprehensive view of all the potential scenarios of the technology. The analysis of consulting companies’ reports also offered a broader vision of future scenarios, thanks to their strategic rather than technical approach [1, 2]. This process led to identify 76 projections that represented the basis for a reflection during the expert face-to-face interviews. After screening the relevant articles and reports, a first filtering of the identified 76 projections was made in order to dismiss redundant or incomplete projections, and to keep only the most complete and varied ones. This process reduced the number of projections to 33 and to 20 after the review of two experts.

### 3.3 The Delphi projections

The formulation of the projections represents the most sensitive part of the research since it influences the whole study. A detailed analysis was carried out in order to avoid mistakes and confusion. In order to facilitate the respondents filling the questionnaire and to avoid any kind of ambiguity, an introduction explaining the meaning of the terminology used in the questionnaire was presented before starting the survey. The developed scenarios were broken down into six macro categories (the same as proposed by Heiko and Darkow [46]) to guarantee a more complete and systemic view of how the blockchain ecosystem and community can change and shape the future. The choice of 20 projections to be evaluated by experts is in line with prior studies exploiting the Delphi method [46, 47]. Parente and Anderson-Parente [47] have proposed to limit the number of Delphi questions (e.g. to 25 questions) in order to guarantee a high response rate and properly filled-in questionnaires, including only closed answers. We decided to add the possibility to comment the given answers in order to gather additional qualitative data to improve the quality of the results, in line with the methodology proposed by Heiko and Darkow [46].

### 3.4 Selection of the panel of experts

As blockchain experts that took part to the survey, we selected individuals working in companies and institutions on the basis of their experience and knowledge of the field. Following Adler and Ziglio [48] and Heiko and Darkow [46] four requirements for “expertise” were considered:

- knowledge and experience on blockchain technology;
- capacity and willingness to participate to the Delphi study;
- sufficient time to participate to the Delphi study;
- effective communication skills.

**Fig 3:**
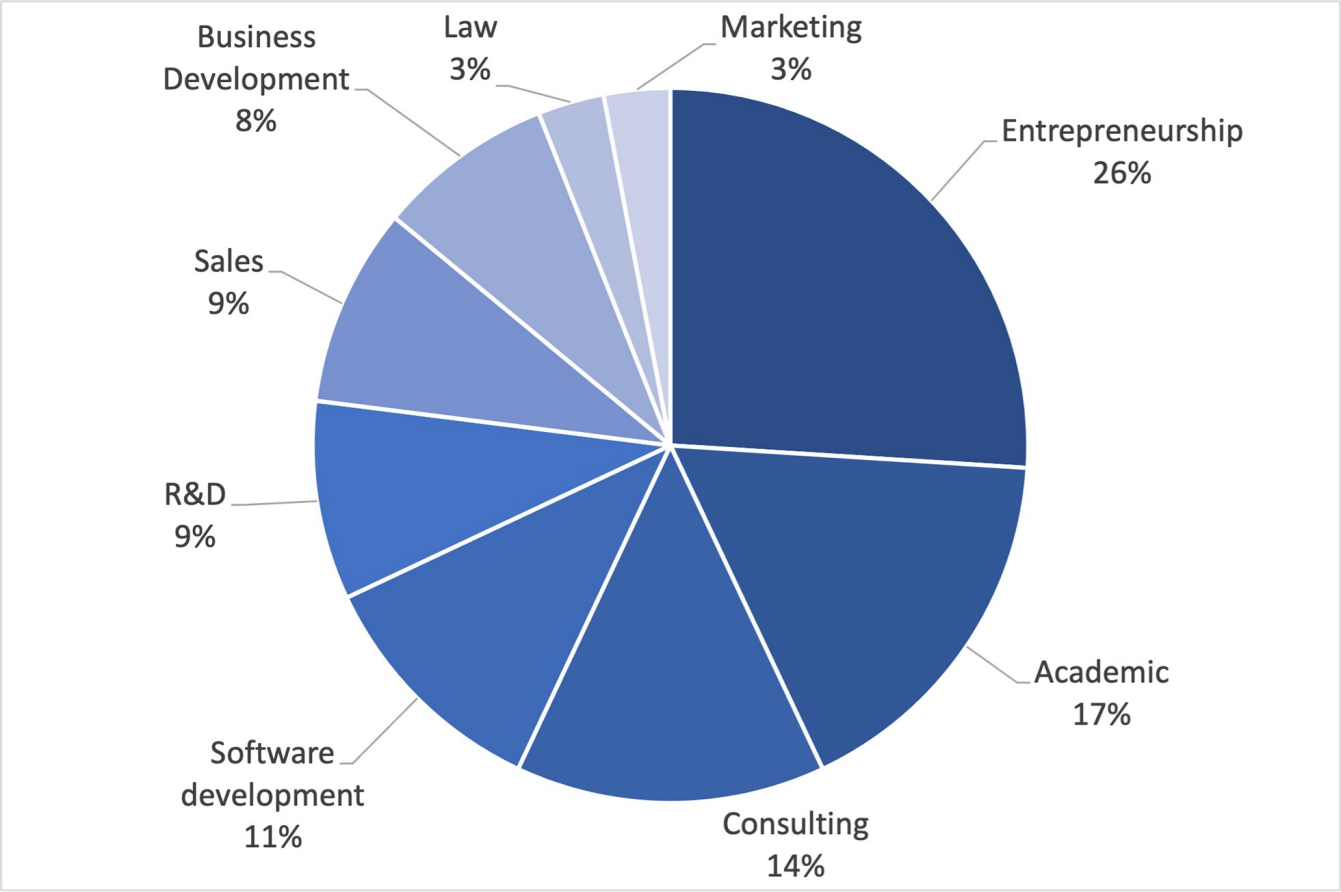
Experts’ backgrounds.

**Fig 4:**
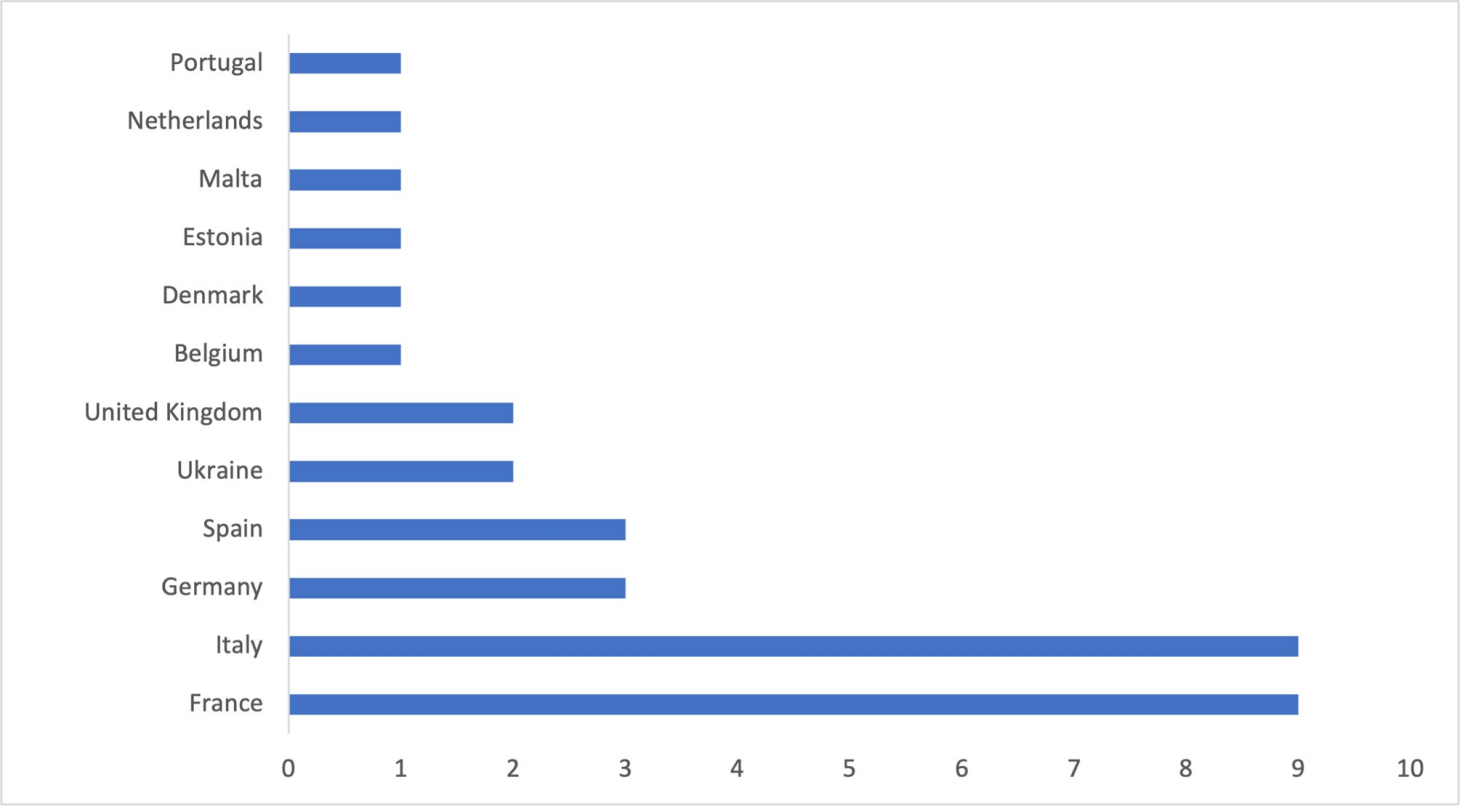
Geographical distribution of the experts.

**Fig 5:**
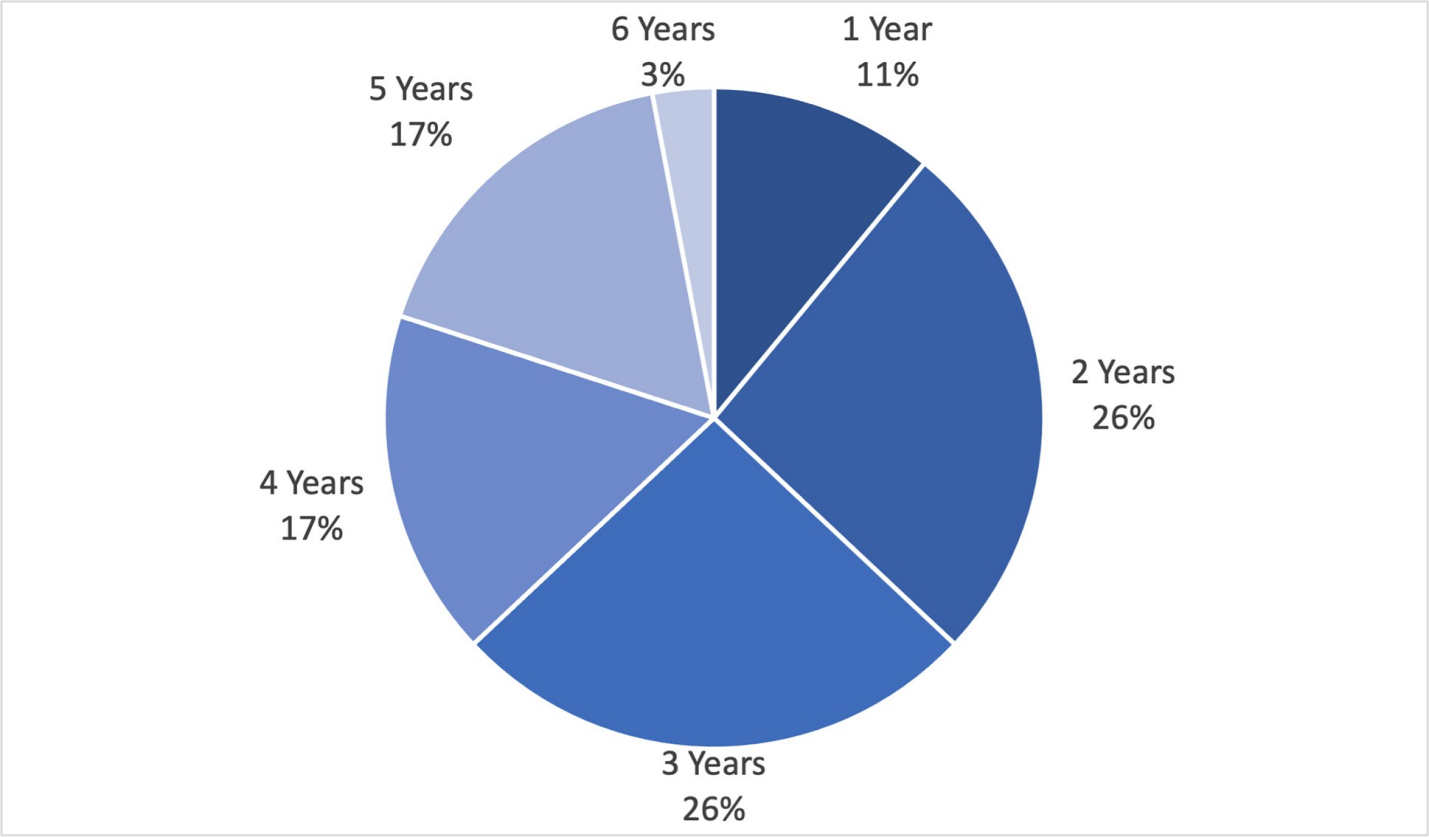
Experts’ working experience seniority.

A minimum panel size of 15–25 participants is often required to lead to consistent results. In our case, a panel of 35 experts was reached for the first round. For the reliability of the study the panelists were selected with different backgrounds and profiles. To be aligned with the European focus of the study, we considered experts working in twelve European countries, being France and Italy the ones with the highest number of respondents. The panel characteristics are reported in Figs 3, 4 and 5.

### 3.5 Execution of the Delphi surveys

In line with the methodology proposed by Heiko and Darkow [46], two rounds of surveys were executed. We decided to carry no more than two rounds because participating to a Delphi study requires a lot of effort and is a time-consuming task for panelists. By limiting the rounds to two, we reached a sufficient number of respondents that led to have valuable results and consistent conclusions. Moreover, since for each scenario the possibility to include a qualitative argumentation was included, the smaller number of iterations worked as a stimulus for the experts to explain the reasons of their quantitative answers.

The survey was carried out following the standards of the Internet-based Delphi, also called e-Delphi [39, 40]. Giving the possibility to respondents to answer digitally allowed experts to be more flexible in responding to the survey, ensuring a greater participation. The way the questionnaire was structured was exactly as the e-Delphi website suggests, but for practical reasons we edited the survey using Google Form. Other standards, such as the real-time Delphi solution proposed by several studies [14, 42, 43, 49] could have led to a better comparison among experts, but would have likely caused more withdraws to the survey.

#### 3.5.1 First round

In the first round of the survey, the experts assessed the expected probability and impact of the twenty outlined projections. Some Delphi studies [50, 51] include a third factor that helps to assess the desirability of a scenario (i.e. how much an expert is in favour of the realization of a prediction). However, we decided not to include this last aspect to make the questionnaire lighter and faster to be filled in, and to reduce drop-outs (Table 2).

**Table 2:** Survey dropouts during the whole process.

	Survey invitation	First round	Second round
**No. Of experts**	50	35	28
**Percentage**	100%	70%	56%

Impact, evaluated at the industry level, was measured on a five-point Likert scale [52]. Since there is not a general consensus among experts regarding the number of points the scale should have, and due to the general nature of the scenarios, we preferred to use a five-point Likert scale. The corresponding probabilities are: 0%, 25%, 50%, 75% and 100%. Gathering quantitative data allowed to perform a first set of analyses based on descriptive statistics (e.g. mean, median and interquartile range-IQR). We used qualitative data, instead, to build the final scenarios during the fourth step of the forecasting technique. Even though the literature regarding the Delphi method does not suggest a standardized way to analyze consensus, central tendency measures, such as median and mean values, are useful to grasp a first understanding and are frequently accepted and adopted (Table 3). Scenarios with an IQR equal or lower than 1.5 were considered as having reached an acceptable degree of consensus. It should be noticed that most of the projections that achieved the highest probability, having a median value of 75% achieved also the consensus, i.e. IQR below 1.5. This was the case for projections 3, 4, 8, 9, 10, 13, 15, 19, 20.

**Table 3:** Central tendency measures for the first round of surveys.

	*Projections*	*Round 1*		
		*Probability*		*Impact*
		*Median*	*IQR*	*Median*
***Socio Cultural***				
1 -	Education and knowledge	0,5	2,5	4
2 -	European Union as leader hub	0,25	1,5	4
***Policy and Regulations***				
3 -	Security and cost reduction	0,75	1,5	4
4 -	Standards and governance	0,75	1	4
5 -	Regulations will foster innovation	0,5	2	4
***Economic***				
6 -	Intermediaries	0,5	1,5	4
7 -	Financial credit services	0,5	2	4
8 -	Change business model	0,75	1,5	4
9 -	Competitive advantage	0,75	1	4
***Technological***				
10 -	Trace transaction and auditing purposes	0,75	1	4
11 -	Reliability of credit systems	0,5	2	4
12 -	Access to loans	0,5	2	4
13 -	Issue and transfer equity shares	0,75	1,5	4
14 -	Financing through ICOs	0,5	2	4
15 -	Reliable and transparent transactions	0,75	1	5
***Business***				
16 -	Private blockchains	0,5	1,5	3
17 -	Cryptocurrencies	0,5	1	3
18 -	Digitization of shares	0,75	2	4
19 -	Open-source projects	0,75	1	4
20 -	Smart contracts	0,75	1	5

These results show that it was easier for experts to find a consensus over the projections that resulted as very likely to occur. Only projection number 18 achieved a high probability score but could not reach a consensus.

#### 3.5.2 Second round

During the Delphi’s second round only the projections with an IQR above 1.5 (i.e. which did not reach consensus in the first round) were tested. In order to allow the respondents to easily understand the answers that the panel gave as a whole in round one, for each projection a quantitative report was provided. This report was made of a bar chart with the distribution of the first round’s answers and the correspondent qualitative details, i.e. some of the argumentations provided by some of the panelists. Experts were asked to reconsider the likelihood of occurrence of the projections number 1, 5, 7, 11, 12, 14 and 18. The second round was again structured using Google Form. Following the Delphi’s approach, we did not ask again to estimate the impact for each projection, since this would have presumably been not subject to any change. Moreover, we decided to leave the opportunity to offer again some qualitative comments in support of the answers for a better analysis of the results. The number of experts who successfully completed the second round of the survey dropped to 28, i.e. the 80% of the experts that completed Round 1 and 56% of the selected initial panel. Again, we evaluated the central tendency measures for the projections tested during the second round (Table 4).

**Table 4:** Central tendency measures for the second round of surveys.

*Projections*	*Round 2*		
	*Probability*		*Impact*
	*Median*	*IQR*	*Median*
***Socio Cultural***			
Education and knowledge	0,25	1,25	4
European Union as leader hub	-	-	-
***Policy and Regulations***			
Security and cost reduction	-	-	-
Standards and governance	-	-	-
Regulations will foster innovation	0,75	1,25	4
***Economic***			
Intermediaries	-	-	-
Financial credit services	0,50	2	4
Change business model	-	-	-
Competitive advantage	-	-	-
***Technological***			
Trace transaction and auditing purposes	-	-	-
Reliability of credit systems	0,75	1,25	4
Access to loans	0,37	2	4
Issue and transfer equity shares	-	-	-
Financing through ICOs	0,50	2	4
Reliable and transparent transactions	-	-	-
***Business ***			
Private blockchains	-	-	-
Cryptocurrencies	-	-	-
Digitization of shares	0,75	1	4
Open-source projects	-	-	-
Smart contracts	-	-	-

## 4 Results

In order to provide a more effective and structured analysis of the results, we first report the final summary table of the Delphi survey and then describe the insights obtained from the analysis. It has to be noticed that Table 5 reports quantitative data only, while during the survey qualitative data were collected as well. In presenting the results of this research, both quantitative and qualitative data are used to provide the best possible picture of what the blockchain-based future will look like. Alongside with standard statistics, we build on qualitative insights obtained during the interviews carried on with experts.

**Table 5:** Quantitative data obtained during the two rounds of surveys.

	*Projections*	*Round 1*		*Round 2*		* *
		*Probability*		*Probability*		*Impact*
		*Median*	*IQR*	*Median*	*IQR*	*Median*
***Socio Cultural***						
1 -	Education and knowledge	50%	2,5	25%	1,25	4
2 -	European Union as leader hub	25%	1,5	-	-	4
***Policy and Regulations***						
3 -	Security and cost reduction	75%	1,5	-	-	4
4 -	Standards and governance	75%	1	-	-	4
5 -	Regulations will foster innovation	50%	2	75%	1,25	4
***Economic***						
6 -	Intermediaries	50%	1,5	-	-	4
7 -	Financial credit services	50%	2	50%	2	4
8 -	Change business model	75%	1,5	-	-	4
9 -	Competitive advantage	75%	1	-	-	4
***Technological***						
10 -	Trace transaction and auditing purposes	75%	1	-	-	4
11 -	Reliability of credit systems	50%	2	75%	1,25	4
12 -	Access to loans	50%	2	37,5%	2	4
13 -	Issue and transfer equity shares	75%	1,5	-	-	4
14 -	Financing through ICOs	50%	2	50%	2	4
15 -	Reliable and transparent transactions	75%	1	-	-	5
***Business***						
16 -	Private blockchains	50%	1,5	-	-	3
17 -	Cryptocurrencies	50%	1	-	-	3
18 -	Digitization of shares	75%	2	75%	1	4
19 -	Open-source projects	75%	1	-	-	4
20 -	Smart contracts	75%	1	-	-	5

Firstly, it is interesting to analyze which projections, out of the initial 20, reached a significant consensus (IQR <1.5 after the two rounds of the surveys) and were given a high probability of occurrence by the experts. We can summarize the findings in this domain around three major axes: efficiency, security, and innovation.

By 2030, it will be easier, faster and leaner to exchange value and data among users, institutions and countries. Efficiency will boost and uncover innovation potential within companies and societies if these latter will be able to exploit such a new opportunity. Policies will be a necessary pre-requisite for companies to be able to build a competitive edge globally. From this perspective, the capability of central governments to spur innovation with lean and flexible regulations will be a key driver in explaining the ex-post productivity differential among companies belonging to different countries. From the interview with an investment banker part of the BPCE French group (one of the largest banks in France), it emerged how efficiency is often hampered by the lack of an equally efficient regulation. To provide the reader with an interesting example, in 2018, Natixis, the international corporate and investment banking, asset management, insurance and financial services arm of BPCE, entered the Marco Polo consortium, an initiative born to provide a newly conceived trade and supply chain finance platform, leveraging Application Programming Interfaces (APIs) and blockchain technology. Many other leading banks joined the consortium as well. However, as highlighted by the investment banker, the main limiting factor of the consortium, strongly hampering its efficiency and ability to provide a competitive edge, was the “old-style” bureaucracy linked to it. Although transactions were in principle to be executed smoothly, a bulk of legal paperwork was required to approve them formally. In this case, it appears evident that technology often runs faster than policy, consistently lowering its potential. Interestingly, this view is also shared by regulatory bodies. An experienced lawyer and notary, also member of a panel of experts elected by the Italian government to define the national strategy on blockchain, highlighted that, sometimes, regulators working on blockchain-related policies are trying to adapt existing regulations to the new paradigm. Due to the intrinsically different nature of the technology, this could represent a wrong approach. At the same time, building a new set of policies from scratches could represent a challenging task. From this perspective, projections 4 and 5 confirm this insight: policy and technology should come hand in hand to synergically boost productivity. The three projections reached consensus after the two rounds and were assigned a high probability of occurrence. Overall, it is evident that regulatory aspects linked to the adoption of this new technology shall not be underestimated.

As previously mentioned, security, and specifically cybersecurity, is another dimension around which blockchain could bring consistent advantages, as projections 3, 10, 11 and 15 suggest. On this specific aspect, we interviewed a project leader of the World Economic Forum who previously worked for the United Nations for more than ten years. She dealt specifically with digital regulations, justice, and cybersecurity, and in the last three years before the interview, she specifically worked on blockchain implications and how the technology could be implemented in existing ecosystems. Thanks to her experience in the domain, she clearly explained how the blockchain represents a meaningful technology to avoid cyberattacks to sensitive data and digital files. In her opinion, the avoidance of a single point of failure is the main reason behind a possible blockchain adoption over the next years, since cyberattacks are becoming more frequent and dangerous and related costs for companies are exponentially increasing (e.g. 2020 has been a record year for cyber attacks). Consequently, companies will be increasingly investing in distributed ledgers as a form of contingency budget to lower the cybersecurity risk and its related cost. Given the centrality of data in today’s businesses, serious attacks and loss of data could represent a serious threat to business long-term sustainability.

The third relevant aspect on which blockchain will have a strong impact is, not surprisingly, innovation. Although regulation could represent a non-negligible limiting factor, experts foresee many sectors to be impacted by the technology adoption. For example, the financial sector could be heavily affected by this new paradigm. Particularly, companies’ capital structures and their strategic interlink with business models will drive a differential competitive power. Most likely, enterprises will have to rethink their business models to account for the possibility to digitize/tokenize their assets (Projections 8 and 18). The capability in flexibly adapting their service offerings to the new opportunity and the ability to raise, and re-invest, new capitals will shape the global competition landscape across different industrial sectors and geographies. From one side, blockchain will enable new strategic decisions, from the other side, it will be of fundamental importance to build technological capabilities to enable these decisions. The underlying technology behind transactions, equity offering and equity share transfers will most likely be the blockchain (Projections 13 and 16). Disintermediation and the ability to exchange value, information, and data trustfully without a central authority will enable a new way of funding and cooperation on open-source projects (Projection 19). Most likely, people will refer to blockchain systems as they now refer to browsers such as Chrome, Firefox or Internet Explorer. Many blockchains are already available and are constantly improved and developed, and it is foreseeable that this will remain the case in the future. Users will just need to know the characteristics that a blockchain provides to choose the most suitable one for their business and purposes. Blockchain-based systems will require new skills and knowledge that developers and engineers will need to develop. Big efforts will be needed to make the blockchain more and more user friendly and attractive for those who just want to benefit from the immutability, traceability, and security that it intrinsically brings. At the time of the writing and in line with the Abernathy and Utterback model [53] many players are currently investing and innovating on blockchain to provide services that will satisfy the new market needs.

The opportunity for people to deal freely will in fact generate opportunities that were unforeseeable before. Self-enforcing smart contracts (Projection 20) will let parties to buy and sell products or to rent them with pay-for-use schemes in an automated way, the digitization of shares and assets will allow companies to raise capital in new ways, without the need to rely on banks, venture capitals or traditional IPOs. Indeed, it is important to understand how the digitization of assets can challenge existing investments and the funding industry represented by traditional private equity firms and banks. Blockchain could allow the creation of platforms for the issuance of traditional financial products on a tokenized nature, making it easier, more transparent and cheaper to manage and access these tools for everyone, including both individual savers and SMEs. Two different types of companies can and will operate in the market: those which have blockchain at their core since their foundation, and those which have (or will have) to embark in a digital transformation process to reconvert themselves into blockchain-based enterprises. In both cases, companies are investing to get a competitive advantage over competitors, betting on the technology that is promising to reduce costs and increase efficiency. Once a dominant design in product and services will be achieved, companies that took a different path will likely exit the market, letting firms following the dominant design to gain market shares.

To conclude and to conceptualize the insights we obtained from both quantitative and qualitative data, we derived four scenarios that we organized in a matrix framework, reported in Table 6. The framework was built around two main dimensions: on one hand the digitization of assets, and on the other hand the change in business models. The proposed framework leads to the identification of four quadrants: scenarios which envision both the digitization of assets and business model changes and scenarios which do not foresee neither of these two changes. These four main development scenarios were completed and analyzed in the light of the conducted interviews and of the quantitative and qualitative data gathered through the Delphi survey. Each quadrant was given a label: Internal Processes, Flow-less Coopetition, Suppliers Potential and Investment Opportunities. When discussing the quadrants, we try to highlight which of the three improvement areas previously identified (efficiency, security, and innovation) are exploited in the discussed scenario.

**Table 6:** Final scenarios.

		Assets Digitization	
		No	Yes
**Business Model Change**	**Yes**	**Flowless Coopetition**	**Investment Opportunities**
		• Elimination of information asymmetry	• Autonomous Trading
			• New financial instruments (e.g. STOs)
		• Elimination of intermediaries	
		• Reduction of uncertainty	• Transfer and settle capital
		• Coopetition Paradox	• Token economics
		• Network fee charges and rewards	• Regulation Issues
	**No**	**Internal Processes**	**Supplier Potential**
		• Anytime, anywhere traceability	• BaaS
		• Data reliability and security	• Development platforms and decentralized apps (dApps)
		• Straight-through processing	
			• Blockchain protocols
			• New Exchanges and marketplaces
		• Sharable information	
			• New entrants

To derive relevant insights from the framework, it is useful to start from the bottom left quadrant, Internal Processes. This name was chosen to highlight the absence of any particular evolution for the company at a strategic level through the blockchain adoption. In this case, it is conceivable to use the technology to incrementally improve firms’ operation performances. Blockchain’s main benefits are to increase traceability of transactions and guarantee their immutability. All these characteristics adopted on today’s processes will result in an automation of routine business functions, such as settlements and reconciliation, customs clearance, heavy payments, invoicing, and documentation, boosting operational efficiency and cost performance. In this scenario, security and efficiency will see a consistent improvement.

The top-left scenario shows instead a different perspective, by considering a broader adoption of blockchain that generates new cooperative business models among different stakeholders, potentially even among competitors. This is why it is called Flow-Less Coopetition. In this case, the benefits of blockchain will help at generating a more democratic ecosystem in terms of information. Those actors that base their business models on information asymmetry, having access to key information before others, will need to revisit their business models if they want to stay competitive. It is of interest to notice how big financial institutions, traditionally competing, are now exploring potential collaboration models in the light of this new technology (e.g. JP Morgan Chase, Morgan Stanley). This quadrant envisages an advance in all three blockchain-enabled dimensions: efficiency, security, and innovation.

The bottom-right scenario, called Suppliers Potential, highlights how, thanks to the digitization that blockchain allows, many actors could jump in the market providing solutions to those companies that would like to benefit from the advantages of digitizing their assets, but are lacking means and competences to internally develop them. Those companies would rather outsource the development of blockchain-based solutions. For this reason, the potential for the creation of a remunerative B2B market exists. Even though there are already protocols that are leaders in the market (Hyperledger Fabric and Ethereum), new solutions with different configurations will likely be needed to support different industries and use case solutions. As for the first scenario, also in this context efficiency and security will be mainly affected.

Finally, the last scenario (Investment Opportunities) focuses on the combination between the complete digitization of the assets of a company and the new business models that this major change could generate. As already mentioned in previous paragraphs, industries are experimenting many ways to facilitate the access to capital. Since the explosion of ICOs in 2017, new and easier ways to access capital have become possible and achievable. However, due to their unregulated nature, ICOs still present numerous potential threats (Projection 14 did not reach consensus). For this reason, other solutions, such as STOs (Security Token Offerings), are on the way of being tested. Bringing a higher degree of freedom to investments will allow companies to receive funds from diverse and non-traditional investors, and it will also boost investments by private individuals into early-stage companies. Efficiency and innovation will be at the core of this last scenario.

## 5 Conclusions

In this paper, we studied different blockchain-based projections and we assessed their likelihood and impact thanks to the participation of a pool of experts. We built our findings around three dimensions (efficiency, security, and innovation) and we derived four scenarios based on experts’ shared vision. Being the current literature widely fragmented, we believe this research represents a useful starting for conceptualizing blockchain potential and implications. While many research papers focus on blockchain specific applications or general reviews of the state of the art, we try to propose a unifying framework building on different typologies of insights and analyses. We merged quantitative observations derived from standard statistics with qualitative insights obtained directly from experts’ opinions.

Overall, we believe our research can constitute a useful tool for many practitioners involved in the innovation ecosystem and for managers of small, medium and large enterprises to look at future possible scenarios in a more rational and systematic way. From one side, a company’s management can use these forecasts as a starting point for the implementation of new strategies. As previously highlighted, blockchain offers endless possibilities. However, the ability to focus on activities and projects with a positive return on investment will be crucial. Firstly, managers will face the choice between insourcing or outsourcing the technological development of the platform. While the former choice ensures higher flexibility, it also generates high development and maintenance costs. Companies which will identify blockchain as their core service will be entitled to adopt this first strategy, while the majority of the enterprises will probably gain better competitive advantages adopting Blockchain as a Service (BaaS) solution. This latter approach will boost companies’ performances, by enhancing new service offerings as well as a new level of operational efficiency, without carrying the burden and costs of technological complexity.

As mentioned, we believe this research provides useful insights for policy makers as well. The adoption of blockchain represents a tremendous technological change bringing along interesting and tangible opportunities. However, different threats can be foreseen. Central authorities do not only solve the problem of trust in certifying value transactions. They also provide essential supervision on the process itself, for example ensuring that information asymmetry is kept at reasonable levels between parties engaging in any sort of contracts, especially in the financial world. Letting people directly exchange value between themselves or allowing companies to easily raise capitals can boost financial efficiency, but also provides room for frauds and ambiguous behaviours. Today, companies which are interested in raising capitals both through innovative tools such as crowdfunding or through traditional entities such as public financial markets, have the duty to disclose relevant information and usually go through a deep process of due diligence. Regulators should ensure the same level of control on companies that will raise money through Initial Coin Offerings or other sort of blockchain-enabled offerings. We believe that the first step towards a fair regulation of this newly born technology is the understanding of its foreseeable impact on the society in the near future. This work aims to be a precious enabler in this direction. As highlighted in the body of this research, it appears fundamental for policy makers, regulators and government to deeply understand the potential upsides and threats of this new technology, and to correctly navigate the different possible blockchain-enabled scenarios. The successful cooperation between companies’ management and regulators could enable significant productivity shifts in the economic tissue of many countries. Failing in efficiently grasping and enhancing these new paradigms from a regulatory perspective could result into a heavy deficit for the competitive edge and productivity of the industrial sectors of the governments’ respective countries, potentially leading to macroeconomic differentials in productivity.

To conclude, this research could be a useful reference for orienting into this complex and dynamic environment, reducing the perceived uncertainty associated to such a new technology. Thanks to the experts’ advice, it is now possible to have a clearer picture of the evolution of blockchain technologies and of the opportunities and threats that the technology will generate. Certain limitations and characteristics of this study must be considered to correctly and effectively take advantage of its results. The main objective of this work was to examine the most disrupting aspects that are likely to occur in Europe by 2030, with a particular focus on how the technology will facilitate financing, reduce costs, increase transparency and, in general, influence firms’ business models. From this point of view, the objectives and assumptions presented at the beginning of this paper can be considered as fully achieved, but further works exploring other industries and geographies are required to get an organic understanding of the new enhanced paradigms.

Our research only paves the way for a better understanding of what a blockchain-based future will look like, as the differences between industries are too large to be analyzed in a single work. Organizations and businesses in the financial world are consistently changing, but it will be necessary also for companies belonging to different sectors to completely rethink their core activities. From this perspective, we believe further works are needed in these directions. We hope researchers will use and explode our framework to further characterize and meticulously describe the new possible paradigms around the multiple dimensions examined in this work.

## Supporting information

S1 Data (XLSX)
